# Meat consumption and the risk of general and central obesity: the Shahedieh study

**DOI:** 10.1186/s13104-022-06235-5

**Published:** 2022-11-01

**Authors:** Shaghayegh Khodayari, Omid Sadeghi, Maryam Safabakhsh, Hassan Mozaffari-Khosravi

**Affiliations:** 1 Department of Nutrition, School of Public Health, Shahid Sadoughi University of Medical Sciences, Yazd, Iran; 2 Students’ Scientific Research Center, Tehran University of Medical Sciences, Tehran, Iran; 3 Department of Community Nutrition, School of Nutritional Sciences and Dietetics, Tehran University of Medical Sciences, Tehran, Iran; 4 Department of Nutrition, School of Public Health, Shahid Sadoughi University of Medical Sciences, Yazd, Iran

**Keywords:** Obesity, Body mass index, Waist circumference, Meat, Poultry, Fish

## Abstract

**Objective:**

This study aimed to investigate the relations of total meat intake and its subtypes, including red and processed meat, white meat, poultry, fish, and organ meat to the risk of general/central obesity.

**Methods:**

This cross-sectional study included a total of 7312 Iranian adults with the age range of 35–70 years from the Shahedieh cohort study, Yazd, Iran. Dietary intake of subjects was evaluated using a validated 120-item Food Frequency Questionnaire. General obesity was defined as body mass index ≥ 30 kg/m2 and central obesity as waist circumference ≥ 102 cm in men and ≥ 88 cm in women.

**Results:**

After controlling for potential covariates including energy intake, age, marital status, gender, physical activity, supplement use, house possession, education, family size, current smoking, night shift working, history of thyroid disease and depression, and intakes of vegetables, legumes, nuts, fruits, whole grains, and dairy, a significant direct association was found between the higher consumption of white meat (OR = 1.31; 95% CI: 1.06–1.61) and poultry (OR = 1.23; 95% CI: 1.04–1.45) with odds of general obesity. Processed meat was a significant predictor for central obesity in the fully adjusted model, so that individuals in the fourth quartile of processed meat intake, compared with those in the first quartile, had a 22% (OR = 1.22; 95% CI: 1.04–1.43) increased risk to be centrally obese.

**Conclusion:**

This study reveals that higher intakes of white meat and poultry are associated with increased risk of general obesity, while, processed meat consumption was associated with central obesity.

## Introduction

Obesity is a multifactorial public health crisis featured by an excessive store of adiposity, related to a variety of comorbidities such as cardiovascular diseases, dyslipidemia, type 2 diabetes, and some malignancies [1–5]. Globally, 57.8% of adults are estimated to have obesity by 2030 [6]. The prevalence of obesity is growing at a worrying rate, particularly in Asian regions [7]. Among Iranian adults, obesity prevalence elevated from 12% to 2000 to 22% in 2011 and general and central obesity has been reported in 9.7% and 27.7% of adults, respectively [8]. However body mass index (BMI) is a common criterion used for recognizing obesity, its limitation is that it might overlook some people with obesity [9]. General obesity (BMI ≥ 30 kg/m^2^), estimates body fat mass without considering its distribution, whereas central obesity, reflecting body fat distribution, is a stronger predictor of obesity-derived complications compared with BMI [9]. Nevertheless, preventive approaches targeting improving both general and central obesity allow more precise management of obesity and its health-threatening comorbidities.

It is of great importance to recognize modifiable risk factors of obesity to reduce the burden of obesity and related metabolic diseases. Environmental and genetic factors play role in the etiology of obesity [10]. Diet, as an environmental factor, is one of the most important contributors to the obesity pandemic [11]. Meats are a part of the human diet, which not only provide protein and high-quality nutrients, but also are a main source of saturated fatty acids and cholesterol [8]. A protein-rich diet has been linked to greater weight loss in clinical trials [12]; however, there is evidence showing that meat intake in a regular diet increases the risk of obesity [13]. The particular involvement of meat intake in obesity is unclear. While some studies revealed a direct relationship between red meat intake and obesity [14], others failed to find any associations [15, 16]. The majority of previous investigations have mostly concentrated on red meat, and the relation of white meat to general and central obesity is understudied and available evidence is debatable [17, 18]. Furthermore, most studies in this area of research have been conducted in Western countries where red meat consumption has been reported to be high, while people living in the Middle East, have a special pattern of consumption. Iranian people consume a large amount of carbohydrates in their usual diets [19] and have a higher intake of full-fat red meats, compared to other meats [8]. Moreover, the intake of fish per capita in Iran is lower than the international standard [20]. Therefore, this study aimed to investigate the link between total meat intake and its subtypes, including red meat, poultry, processed meat, and fish to the risk of general and central obesity in an Iranian population.

## Methods

### Participants

This study was an enrolment phase dataset of Shahedieh cohort study, conducted in Yazd, Iran, as a part of the PERSIAN cohort (Prospective Epidemiological Research Studies in Iran). The PERSIAN cohort aimed to investigate the predisposing factors of non-communicable diseases among Iranian adults, which is being performed in various cities of Iran (Ahvaz, Ardabil, Bandar Abbas, Fasa, Guilan, Kermanshah, Mashhad, Mazandaran, Rafsanjan, Sabzevar, Shahrekord, Shiraz (2 sites), Tabriz, Urmia, Yasuj, Yazd, and Zahedan). Detailed characteristics of the PERSIAN cohort have already been published [21]. In summary, initially, all 10,194 adults, aged 35–70 years, living in the Shahedieh district of Yazd, Iran, were invited to participate in the Shahdieh study during 2015–2016. Among them, a total of 9,983 subjects participated in the study. Inclusion criteria were age between 30 and 75 years and residence in the Shahedieh district at least for 9 months each year. People who were physically or mentally disabled were excluded from the study due to not being able to fully complete the study. Individuals were invited to visit the health center of Shahdieh district to obtain the required information through a face-to-face interview. For the present study, after collecting blood samples from subjects, data regarding food intake, physical activity, and socio-demographic characteristics were gathered by expert evaluators. After excluding individuals with incomplete information on food intake and obesity, as well as those who had unusual total daily energy intake (± 3 SD of mean energy intake), a total of 7312 participants remained for this study. A written consent form was obtained from all participants. This study was conducted according to the guidelines laid down in the Declaration of Helsinki and the protocol of the study was Ethics Committee of Shahid Sadoughi University of Medical Sciences, Yazd, Iran (ID: IR.SSU.SPH.REC.1399.218).

### Dietary assessment

The usual food intake of participants was measured by a validated 120-item Food Frequency Questionnaire (FFQ) by trained interviewers [22]. This FFQ was designed based on Iranian food items to assess the frequency and amount of consumption, based on household measures, for each food item over the past 12 months. Then, the daily consumption (grams/day) of each food item was calculated. Daily nutrients intake of all individuals was calculated with the use of the US Department of Agriculture (USDA) national nutrient database [23], modified for Iranian food items. The modified USDA nutrient database covers the nutrients contents of certain Iranian food items, which were not presented in the original USDA database.

### Anthropometric indices

Body weight was assessed in a barefoot condition with light clothes to the nearest 0.1 kg with the use of an electronic digital scale. The height of participant was also evaluated by a standard stadiometer, with a precision nearest to 0.5 cm. BMI was calculated as weight (kg)/ height in meters squared. Waist circumference (WC) was measured as the minimum abdominal circumference between the last gear and elliptical bone to the nearest 0.5 cm. General obesity was defined as BMI ≥ 30 kg/m2 based on the BMI cutoff points for Caucasian. Central obesity was defined as WC ≥ 102 cm in men and ≥ 88 cm in women according to the cutoff points for Caucasians [24]. To decrease measurement error, all anthropometric indices were assessed in the morning in a fasting condition.

### Assessment of other variables

The physical activity of participants was evaluated with the use of the International Physical Activity Questionnaire by interview [25]. Moreover, data regarding age (What is your age?), sex (What is your gender? (Male/Female/Others)), smoking (Have you smoked at least 1 cigarette per day during the last year? (Yes/No)), home ownership (What is your living status? (Owner/Tenant/Lease/Others)), marital status (What is your marital status? (Divorced/Married/Single)), family size (What is your family size?), depression (Do you have a history of depression? (Yes/No)), thyroid disorders (Do you have a history of thyroid disorders? (Yes/No)), working shift (What is your work shift? (Day shift/Night shift/Rotating shift)), and consumption of multivitamin and mineral supplements (Do you use multivitamin/mineral supplements? (Yes/No)) was acquired by a general questionnaire.

### Statistical analysis

Participants were categorized according to the quartile cutoff points of total meat intake. One-way analysis of variance (ANOVA) and Chi-squared tests were applied to assess differences in quantitative and qualitative variables across quartiles of meat intake, respectively. Odds ratio (OR) and 95% confidence interval (CI) for the association of total meat intake, red meat, white meat, processed meat, organ meat, poultry, and fish with the risk of general and central obesity were calculated using the binary logistic regression analysis. In addition to the crude analysis, 3 adjusted models were applied. Model 1 was adjusted for age, energy intake, and gender. Model 2 was adjusted for variables included in model 1 plus physical activity (continuous), marital status (divorced, married, single), supplement use (yes vs. no), house possession (owner vs. non-owner), education (non-university education vs. university graduate), family size (˂4 individuals vs. individuals ≥ 4), current smoking (yes vs. no), night shift working (yes vs. no), and history of thyroid problems (yes vs. no) and depression (yes vs. no). The last model included variables adjusted for in model 2 plus the intakes of vegetables, legumes, dairy, fruits, whole grains, and nuts. In all models, subjects in the first quartile of meat intake were considered as the reference group. To calculate the trend of OR across quartiles of meat intake, the categories of intake were entered as an ordinal variable in the binary logistic regression. All statistical analyses were carried out with the use of SPSS version 18 and P value ˂0.05 was considered significant.

## Results

A total of 7312 subjects were included in this study. The mean age of study participants was 48.50 ± 9.74 years and 58.4% were female. The prevalence of general and central obesity among participants was 66.0% and 45.33%, respectively. The basic characteristics of subjects among different quartiles of total meat intake are presented in Table 1. Participants in the top quartile of total meat intake, compared with those in the lowest category, were more possible to be male, younger, married, smoker, centrally obese, have a large family size, use supplements, and have frequent night work shifts, while, they were less likely to have a university education, depression, and thyroid disorders.

**Table 1:** Demographic characteristics of participants across quartiles of total meat intake

Variables	Quartiles of total meat intake				
	Q1 (n = 1828)	Q2 (n = 1828)	Q3 (n = 1828)	Q4 (n = 1828)	P-value*
Age (year)					
Mean	51.1	48.5	47.69	46.28	< 0.001
SD	10.1	9.5	9.4	8.9	
Gender (female) (%)	71.9	60.7	52.1	37.1	< 0.001
Marital status (married) (%)	90.6	95.1	97.0	95.1	< 0.001
Current smoker (yes) (%)	7.7	11.2	11.1	18.9	< 0.001
Supplement use (yes) (%)	2.2	3.5	3.2	4.0	0.02
Education (university graduate) (%)	38.5	30.5	31	30	< 0.001
Home ownership (owner) (%)	90.3	91.8	92	92.8	0.05
Family size (> 4 people) (%)	54.2	58.9	63	67.5	< 0.001
Frequent night working shift (%)	2.8	4.6	7.3	8.7	< 0.001
Thyroid disorders (%)	15.6	14	12.6	11.7	< 0.001
Depression (%)	20.1	16.6	14.5	11.5	< 0.001
General obesity (%)	64.1	65.5	66.7	67.7	0.11
Central obesity (%)	37.9	42.7	46.6	54.1	< 0.001

Data are presented as mean ± SD or percent

*Obtained from the one-way analysis of variance (ANOVA) or Chi-squared tests, where appropriate

The intake of nutrients and food groups based on the quartiles of total meat intake is reported in Table 2. Individuals in the top quartile of total meat intake had higher consumption of fruits, vegetables, legumes, nuts, whole grains, dairy, daily energy, protein, carbohydrate, total fat, sodium, saturated fatty acid (SFA), caffeine, polyunsaturated fatty acids (PUFA), magnesium, and calcium, compared with those in the lowest category. No significant difference in the intake of fiber was found among quartiles of total meat intake.

**Table 2:** Dietary intakes of selected food groups and nutrients across quartiles of total meant intake

	Quartiles of total meat intake									
	Q1			Q2		Q3		Q4		P-value*
**Food groups (g/d)**	Mean	SD		Mean	SD	Mean	SD	Mean	SD	
Fruits	345.6		259.5	410.4	272.7	443.6	264.6	519	346.6	< 0.001
Vegetables	301.2		165.2	352.1	205	369.4	186.7	395.9	192.9	< 0.001
Whole grains	37.6		73.8	45.7	79	46.3	71.1	59.2	93.3	< 0.001
Legumes	25.3		20.4	30.2	24	33.7	24.3	38.9	32.4	< 0.001
Nuts	37.2		22	49.6	26	59.7	28.1	77.4	42.9	< 0.001
Dairy	248.9		184.8	287.1	179	338	208.4	373.7	243.6	< 0.001
Total meat	23.4		7.6	42.3	4.8	60.7	6	102.3	35.7	< 0.001
White meat	9.1		5.5	14.8	8.3	20	11.2	28.6	22.9	< 0.001
Processed meat	0.6		1.4	1.2	2.7	1.7	3.5	3.3	8	< 0.001
Organ meat	2.1		2.1	3.1	3.1	4.1	4	6.4	9.2	< 0.001
Red meat	11.6		6.7	22.9	9.2	34.7	12.7	63.9	35	< 0.001
poultry	6.8		4.7	10.5	6.9	14	9.4	19.9	19.7	< 0.001
Total fish	2.2		2.8	4.2	4.8	5.9	6.2	8.6	10.3	< 0.001
**Nutrients**										
Energy (kcal)	2800		112.3	3067	1025.3	3195.5	960.8	3508.3	905.4	< 0.001
Protein (g/d)	93.1		42.1	103.5	38.3	110.9	35.6	126	34.3	< 0.001
Carbohydrate (g/d)	463.6		205.9	498.1	188.5	506.2	175.5	540	162	< 0.001
Total fat (g/d)	76.5		26.9	86.5	26.7	93.7	26.7	106.9	28.5	< 0.001
SFA (g/d)	22.37		7.6	25.8	7.8	28.2	8.1	32.7	9.1	< 0.001
PUFA (g/d)	16.4		6.9	18.3	7.1	19.4	6.9	21.7	7.4	< 0.001
Na (mg/d)	5176.8		2482.8	5401.9	2212.8	5501.2	2106	5714	2027.7	< 0.001
Ca (mg/d)	992.6		426.1	1088.6	408.7	1173.7	423.3	1257.7	449.2	< 0.001
Mg (mg/d)	658.2		362.9	687.2	329.4	690.2	304.8	717.3	270.7	< 0.001
Fiber (g/d)	50.1		29.3	51.6	26.4	50.9	24.4	51.1	21.8	0.32
Caffeine (mg/d)	131.9		121.8	142.1	118.6	133.5	127.8	145.4	123.2	< 0.001

Data are presented as mean ± SD

Abbreviations: SFA: Saturated fatty acid; PUFA: polyunsaturated fatty acids

*Obtained from the one-way analysis of variance (ANOVA)

Multivariable-adjusted odds ratio and 95% CI for general and central obesity across quartiles of meat intake are indicated in Tables 3 and 4, respectively. After controlling for potential covariates including age, gender, energy intake, physical activity, marital status, family size, supplement use, house possession, education, current smoking, night shift working, and history of thyroid disease and depression, there was a significant direct association between the intakes of total meat (OR = 1.30; 95% CI:1.11–1.52), white meat (OR = 1.59; 95% CI:1.37–1.85), processed meat (OR = 1.21; 95% CI:1.05–1.38), poultry (OR = 1.49; 95% CI:1.30–1.71), and fish (OR = 1.30; 95%CI:1.12–1.51) with the odds of general obesity. However, after further adjustments for other food groups, this relationship disappeared for total meat, processed meat, and fish intakes, while, the relation between white meat (OR = 1.31; 95% CI: 1.06–1.61) and poultry intakes (OR = 1.23; 95%CI: 1.04–1.45) with the odds of general obesity remained significant (Table 3).

**Table 3:** Odds ratio and 95% CI for general obesity across quartiles of total and subtypes of meat intake

		Quartiles of meat intake							
		Q1	Q2		Q3		Q4		P-trend*
Total meat		Reference	OR	95%CI	OR	95%CI	OR	95%CI	
	Crude	1.00	0.93	(0.81–1.07)	0.89	(0.77–1.02)	0.85	(0.74–0.97)	0.01
	Model 1	1.00	1.07	(0.93–1.23)	1.12	(0.97–1.29)	1.26	(1.08–1.47)	0.002
	Model 2	1.00	1.08	(0.93–1.24)	1.11	(0.96–1.29)	1.30	(1.11–1.52)	0.001
	Model 3	1.00	0.99	(0.86–1.15)	0.97	(0.83–1.20)	1.00	(0.83–1.20)	0.95
Red meat									
	Crude	1.00	0.99	(0.87–1.13)	0.86	(0.75–0.99)	0.80	(0.69–0.92)	< 0.001
	Model 1	1.00	1.05	(0.92–1.20)	0.98	(0.84–1.13)	1.01	(0.87–1.17)	0.87
	Model 2	1.00	1.03	(0.90–1.19)	0.97	(0.84–1.13)	1.02	(0.87–1.19)	0.98
	Model 3	1.00	1.03	(0.89–1.18)	0.95	(0.82–1.11)	0.98	(0.84–1.15)	0.60
White meat									
	Crude	1.00	1.00	(0.87–1.15)	1.05	(0.91–1.20)	1.10	(0.95–1.26)	0.13
	Model 1	1.00	1.11	(0.96–1.28)	1.33	(1.15–1.53)	1.59	(1.37–1.84)	< 0.001
	Model 2	1.00	1.10	(0.95–1.27)	1.29	(1.11–1.50)	1.59	(1.37–1.85)	< 0.001
	Model 3	1.00	1.06	(0.91–1.23)	1.18	(1.00-1.38)	1.31	(1.06–1.61)	0.007
Processed meat									
	Crude	1.00	0.96	(0.75–1.22)	0.92	(0.82–1.04)	0.95	(0.84–1.07)	0.62
	Model 1	1.00	1.03	(0.80–1.32)	1.05	(0.92–1.20	1.25	(1.09–1.43)	0.01
	Model 2	1.00	1.03	(0.80–1.33)	1.03	(0.90–1.18)	1.21	(1.05–1.38)	0.04
	Model 3	1.00	1.04	(0.8–1.34)	1.01	(0.8–1.16)	1.11	(0.96–1.28)	0.49
Organ meat									
	Crude	1.00	1.00	(0.87–1.15)	1.02	(0.89–1.17)	1.02	(0.89–1.17)	0.94
	Model 1	1.00	1.04	(0.90–1.20)	1.12	(0.97–1.29)	1.14	(0.98–1.32)	0.23
	Model 2	1.00	1.03	(0.89–1.19)	1.10	(0.95–1.28)	1.16	(1.00-1.35)	0.16
	Model 3	1.00	1.02	(0.88–1.18)	1.06	(0.92–1.23)	1.03	(0.88–1.21)	0.85
Poultry									
	Crude	1.00	0.99	(0.87–1.13)	1.04	(0.85–1.26)	1.08	(0.95–1.23)	0.46
	Model 1	1.00	1.16	(1.02–1.33)	1.09	(0.89–1.34)	1.50	(1.31–1.72	< 0.001
	Model 2	1.00	1.14	(0.99–1.30)	1.09	(0.89–1.33)	1.49	(1.30–1.71)	< 0.001
	Model 3	1.00	1.08	(0.94–1.24)	0.99	(0.80–1.22)	1.23	(1.04–1.45)	0.02
Fish									
	Crude	1.00	0.88	(0.77–1.01)	0.93	(0.81–1.06)	0.97	(0.84–1.11)	0.34
	Model 1	1.00	0.97	(0.84–1.12)	1.13	(0.98–1.31)	1.34	(1.16–1.55)	< 0.001
	Model 2	1.00	0.95	(0.82–1.10)	1.11	(0.96–1.29)	1.30	(1.12–1.51)	< 0.001
	Model 3	1.00	0.92	(0.80–1.07)	1.04	(0.89–1.20)	1.09	(0.93–1.29)	0.21

Data are presented as OR (95% CI)

Model 1: adjusted for age, gender and energy intake

Model 2: adjusted for variables included in model 2 plus physical activity (continuous), marital status (married vs. single vs. divorced), supplement use (yes vs. no), house possession (owner vs. non-owner), education (university graduate vs. non-university education), family size (≥ 4 vs. ˂4 people), current smoking, night shift working, and history of thyroid problems (yes vs. no) and depression (yes vs. no)

Model 3: adjusted for variables included in model 3 plus the intake of vegetables, fruits, whole grains, legumes, nuts, and dairies

*Obtained from the binary logistic regression

**Table 4:** Odds ratio and 95% CI for central obesity across quartiles of total and subtypes of meat intake

		Quartiles of meat intake							
		Q1	Q2		Q3		Q4		P-trend*
Total meat		Reference	OR	95%CI	OR	95%CI	OR	95%CI	
	Crude	1.00	0.81	(0.71–0.93)	0.69	(0.61–0.79)	0.51	(0.45–0.58)	< 0.001
	Model 1	1.00	1.18	(1.00-1.38)	1.30	(1.10–1.53)	1.41	(1.18–1.67)	< 0.001
	Model 2	1.00	1.17	(0.99–1.38)	1.27	(1.07–1.51)	1.41	(1.18–1.68)	< 0.001
	Model 3	1.00	1.10	(0.93–1.30)	1.14	(0.96–1.36)	1.14	(0.93–1.40)	0.15
Red meat									
	Crude	1.00	0.95	(0.83–1.08)	0.76	(0.67–0.87)	0.60	(0.52–0.69)	< 0.001
	Model 1	1.00	1.15	(0.98–1.34)	1.10	(0.93–1.30)	1.31	(0.95–1.33)	0.30
	Model 2	1.00	1.13	(0.96–1.32)	1.07	(0.90–1.27)	1.11	(0.94–1.32)	0.45
	Model 3	1.00	1.12	(0.95–1.31)	1.05	(0.88–1.24)	1.08	(0.90–1.28)	0.54
White meat									
	Crude	1.00	0.83	(0.72–0.94)	0.71	(0.62–0.81)	0.62	(0.54–0.71)	< 0.001
	Model 1	1.00	1.05	(0.89–1.23)	1.26	(1.07–1.48)	1.50	(1.27–1.78)	< 0.001
	Model 2	1.00	1.03	(0.87–1.22)	1.20	(1.01–1.41)	1.50	(1.26–1.78)	< 0.001
	Model 3	1.00	0.99	(0.84–1.18)	1.09	(0.91–1.31)	1.23	(0.97–1.55)	0.07
Processed meat									
	Crude	1.00	1.02	(0.81–1.29)	0.79	(0.70–0.89)	0.71	(0.63–0.80)	< 0.001
	Model 1	1.00	1.21	(0.91–1.61)	1.01	(0.88–1.17)	1.34	(1.15–1.56)	< 0.001
	Model 2	1.00	1.19	(0.89–1.58)	1.00	(0.86–1.16)	1.31	(1.12–1.53)	0.002
	Model 3	1.00	1.19	(0.89–1.59)	0.99	(0.85–1.15)	1.22	(1.04–1.43)	0.003
Organ meat									
	Crude	1.00	1.04	(0.91–1.19)	0.93	(0.81–1.06)	0.83	(0.73–0.95)	0.002
	Model 1	1.00	1.24	(1.05–1.45)	1.24	(1.06–1.46)	1.32	(1.12–1.56)	0.001
	Model 2	1.00	1.22	(1.03–1.43)	1.23	(1.04–1.45)	1.33	(1.12–1.57)	0.001
	Model 3	1.00	1.20	(1.02–1.42)	1.18	(1.00-1.42)	1.19	(1.00-1.42)	0.06
Poultry									
	Crude	1.00	0.76	(0.67–0.86)	0.95	(0.79–1.15)	0.64	(0.56–0.72)	< 0.001
	Model 1	1.00	1.12	(0.96–1.30)	1.10	(0.88–1.39)	1.37	(1.18–1.60)	< 0.001
	Model 2	1.00	1.09	(0.94–1.27)	1.07	(0.85–1.35)	1.35	(1.15–1.58)	0.001
	Model 3	1.00	1.03	(0.88–1.20)	0.97	(0.77–1.23)	1.09	(0.90–1.32)	0.72
Fish									
	Crude	1.00	0.75	(0.65–0.85)	0.71	(0.62–0.81)	0.63	(0.55–0.72)	< 0.001
	Model 1	1.00	0.90	(0.76–1.06)	1.14	(0.97–1.35)	1.41	(1.19–1.66)	< 0.001
	Model 2	1.00	0.88	(0.74–1.04)	1.13	(0.95–1.34)	1.36	(1.15–1.61)	< 0.001
	Model 3	1.00	0.86	(0.72–1.01)	1.07	(0.90–1.26)	1.18	(0.98–1.43)	0.004

Data are presented as OR (95% CI)

Model 1: adjusted for age, gender and energy intake

Model 2: adjusted for variables included in model 2 plus physical activity (continuous), marital status (married vs. single vs. divorced), supplement use (yes vs. no), house possession (owner vs. non-owner), education (university graduate vs. non-university education), family size (≥ 4 vs. ˂4 people), current smoking, night shift working, and history of thyroid problems (yes vs. no) and depression (yes vs. no)

Model 3: adjusted for variables included in model 3 plus the intake of vegetables, fruits, whole grains, legumes, nuts, and dairies

*Obtained from the binary logistic regression

In terms of central obesity, we found that a higher consumption of total meat (OR = 1.41; 95% CI: 1.18–1.68) and its specific types including white meat (OR = 1.50; 95% CI: 1.26–1.78), processed meat (OR = 1.31; 95% CI: 1.12–1.53), organ meat (OR = 1.33; 95% CI: 1.12–1.57), poultry (OR = 1.35; 95% CI: 1.15–1.58), and fish (OR = 1.36; 95% CI: 1.15–1.61) was associated with increased odds of central obesity after controlling for potential confounders expect for other food groups (model 2). Nevertheless, after further adjustment for other food groups, this association remained significant only for processed meat (OR = 1.22; 95% CI: 1.04–1.43).

## Discussion

There are some literature about the relation of meat intake to obesity; nevertheless, the extent and nature of these associations have not been recognized. The present investigation aimed to study the relation of different types of meat with the odds of obesity in an Iranian population. We revealed a significant direct relationship between the consumption of poultry and white meat with the risk of general obesity in the fully adjusted model, while, higher intakes of organ meat, red and processed meat, and fish were not related. Furthermore, after controlling for potential covariates, processed meat was significantly linked to the elevated odds of central obesity. No such relationship was identified between the consumption of red meat, fish, and organ meat with general obesity.

The prevalence of obesity in different regions of Iran has been broadly different. The prevalence of general and central obesity in our study was 66.0% and 45.33%, respectively. The prevalence of central obesity in the Persian Guilan cohort study (Guilan site/Some’e Sara) on 10,520 individuals aged 35–70 years was 75.8% [26]. In another study general and central obesity have been reported in 9.7% and 27.7% of adults, respectively [8]. While, an epidemiological update on the prevalence of obesity, reported a prevalence of 22.3% and 31.1% for general and central obesity in adults, respectively [27]. It is important to be considered that the mean daily energy intake of the present population (3142.7 kcal/day) was remarkably high, compared with other studies on the Iranian population [28], which may be related to a high prevalence of obesity observed in the studied population.

The relation of red meat intake to general/central obesity is not well-identified; Previous investigations have identified contradictory findings, including positive [29, 30] or null [16, 31] relationships in this regard. In contrast to our study, a cohort study by Wagemakers et al. found a significant link between red meat intake and central obesity in British adults [15]; The relative intake of red meat in the study by Wagemakers et al. [15] was higher than the intake of red meat in the present study. Moreover, higher intake of red meat was linked to higher odds for central obesity in an Iranian study, but, in line with our study, such an association was not observed for general obesity [8]. Moreover, Montonen et al. showed that people with higher red meat intake have greater BMI and WC [14]. In a population-based, prospective cohort study on Chinese adults, BMI did not differ according to the categories of red meat intake in women, but higher consumption of fatty fresh red meat was related to a higher risk of central obesity; although, no significant relationship with lean red meat intake was observed, which might be resulted from its higher amount of saturated fats, energy, heme iron, and protein, compared with lean red meat [32, 16]. Contradictory findings of studies on the relation between red meat and obesity may be due to differences in adjustment for potential covariates, particularly for other food groups, health status, genetic and cultural background of participants, the difference in cooking, the difference in measurement of WC sites for assessment of central obesity, and the lack of standard definition for red meat, which all make it difficult to compare findings of studies. Thus, further investigations are needed to confirm these findings [33].

This study revealed a significant direct association between the intakes of white meat/poultry and the risk of general obesity. In contrast to our finding, no significant link was identified between white meat intake and general obesity in an Iranian population [8]. While, Vergnaud et al. [18] showed that higher consumption of poultry is linked to a lower risk for general obesity; however, the relative intake of meat was higher than the meat intake of the present study participants. In line with our study, findings from Maskarinec et al. [34] and Oxford Vegetarian Study [35] reported a significant positive relationship between intakes of white meat and general obesity. Moreover, in a multi-ethnic Asian adult population with a restively lower intake of poultry in comparison with poultry intake of our participants [36], it was found that poultry with skin is directly associated with weight gain, while, poultry without skin was not related to weight gain. Nonetheless, the underlying mechanism for this association is unclear [37–40]. Heterogeneity in the previous findings on the relation of white meat/poultry to obesity may be described by differences in sex and age of participants, various methods of cooking and processing of white meat, or intake of different types of white meat such as poultry or fish in various cultures.

The findings of this study showed that processed meat is linked to higher odds of central obesity. The study by Halkjær et al. [33], which assessed nutritional prognosticators of 5-year alteration in WC of Danish participants, in agreement with our findings, showed that a diet high in processed meat was a significant predictor of subsequent WC gain. In a Japanese-Brazilian population, which was at risk of cardiometabolic diseases, higher consumption of processed meat was related to central obesity in men, but not in women [41]. Furthermore, a study on British adults [15], in which the intake of processed meat was relatively higher than the intake of the our participants, a significant link was identified between processed meat intake and central obesity. Moreover, a meta-analysis revealed that processed meat consumption is directly related to an increase in WC [42]. Mechanistically, processed meat contains higher fat and energy but a lower percentage of protein, and can contain twice times the amount of nitrosamine, and 4 times higher sodium amounts, compared with unprocessed meat [43], which both are obesogen [44, 45]. Besides, some investigations have suggested that this association might be mediated by a high content of saturated fatty acids, heterocyclic amines (HAs), polycyclic aromatic hydrocarbons (PAHs), and advanced glycation end products (AGEs) produced during cooking, heating, or processing of meat [46–48]. Also, high consumption of saturated fat in processed red meat could result in weight gain [49]. Evidence suggests that, compared with vegetable sources of unsaturated fats, saturated fats from animal sources have less thermogenic effects [50] and induce more adiposity [49]. As an alternative explanation, processed meat intake is associated with a change in gut microbiota, and an unfavorable composition in gut flora has been shown to contribute to obesity [8, 51]. These mechanisms might justify the increased odds of central obesity associated with the high consumption of processed meat observed in our study [42, 52].

## Conclusion

In conclusion, this study suggests that a diet high in poultry and white meat is positively associated with the odds of general obesity, while a diet high in processed meat is related to elevated odds of central obesity. Additional studies, especially with a prospective design, are needed to confirm our results. Moreover, examining whole dietary patterns in association with obesity provides a better approach than focusing on single food items because of reducing the co-linearity issue which may occur when evaluating single foods [53–56]. This matter should be taken in consideration into future investigations in this field.

## Limitation

This study has some strengths such as a broad range of age for participants, a large sample size, and controlling the results for a comprehensive range of dietary and non-dietary covariates. Most importantly, it has been identified that a higher intake of nuts, vegetables, legumes, fruits, whole grains, and dairies is related to reduced odds of obesity [57, 58]; we adjusted these potential covariates to reach an independent relationship between meat intake and obesity. Notably, we did not control analyses for saturated fat and total fat as they may be mediating rather than confounding variables [16]. We also obtained questionnaire-based data through a face-to-face interview to increase the precision of the information. Nevertheless, when interpreting the results, some limitations of this study should be considered. First, because of the cross-sectional nature of this study, causality could not be inferred. Accordingly, these results are essential to be confirmed by prospective cohort studies. Second, this study did not evaluate the types of red meat (full fat, lean, and low fat). However, people living in Iran often consume full-fat red meat. Thus, red meat in the present study could be considered a high-fat, high-energy food item in Iran. Finally, similar to other epidemiological studies, the random error in reporting food intake is an important limitation; although, a validated FFQ was applied for the evaluation of food intake.

## Data Availability

Data can be reached by contacting the corresponding author.

## Abbreviations

**OR**: Odds ratio
**CI**: Confidence interval
**BMI**: Body mass index
**SD**: Standard deviation
**FFQ**: Food frequency questionnaire
**USDA**: US Department of Agriculture
**WC**: Waist circumference
**ANOVA**: Analysis of variance
**SFA**: Saturated fatty acid
**PUFA**: Polyunsaturated fatty acids
**CVD**: Cardiovascular disease
**HAs**: Heterocyclic amines
**PAHs**: Polycyclic aromatic hydrocarbons
**AGEs**: Advanced glycation end products
